# Evaluation and Characterization of Curcumin-β-Cyclodextrin and Cyclodextrin-Based Nanosponge Inclusion Complexation

**DOI:** 10.3390/polym13234073

**Published:** 2021-11-24

**Authors:** Hadeia Mashaqbeh, Rana Obaidat, Nizar Al-Shar’i

**Affiliations:** 1 Department of Pharmaceutical Technology, Faculty of Pharmacy, Jordan University of Science and Technology, Irbid 22110, Jordan; 2 Department of Medicinal Chemistry, Faculty of Pharmacy, Jordan University of Science and Technology, Irbid 22110, Jordan; nashari@just.edu.jo

**Keywords:** cyclodextrin-based nanosponge, cross-linking, complexation stability

## Abstract

Cyclodextrin polymers and cyclodextrin-based nanosponges have been widely investigated for increasing drug bioavailability. This study examined curcumin’s complexation stability and solubilization with β-cyclodextrin and β-cyclodextrin-based nanosponge. Nanosponges were prepared through the cross-linking of β-cyclodextrin with different molar ratios of diphenyl carbonate. Phase solubility experiments were conducted to evaluate the formed complexes and evaluate the potential of using β-cyclodextrin and nanosponge in pharmaceutical formulations. Furthermore, physicochemical characterizations of the prepared complexes included PXRD, FTIR, NMR, and DSC. In addition, in vitro release studies were performed for the prepared formulations. The formation of β-cyclodextrin complexes enhanced curcumin solubility up to 2.34-fold compared to the inherent solubility, compared to a 2.95-fold increment in curcumin solubility when loaded in β-cyclodextrin-based nanosponges. Interestingly, the stability constant for curcumin nanosponges was (4972.90 M^−1^), which was ten times higher than that for the β-cyclodextrin complex, where the value was 487.34 M^−1^. The study results indicated a decrease in the complexation efficiency and solubilization effect with the increased cross-linker amount. This study’s findings showed the potential of using cyclodextrin-based nanosponge and the importance of studying the effect of cross-linking density for the preparation of β-cyclodextrin-based nanosponges to be used for pharmaceutical formulations.

## 1. Introduction

Curcumin is a naturally occurring compound isolated from Curcuma longa, and it earned substantial interest over the past few decades owing to its beneficial effects on human health [1,2]. 

Curcumin has a wide range of therapeutic properties, including antioxidant [3], antimicrobial [4], anti-inflammatory [5], anti-viral [6], and neuroprotective properties [7]. In addition, various studies have also reported the effect of curcumin on apoptosis induction and the inhibition of proliferation in various kinds of tumorous cell lines, such as human colorectal [8,9], liver [10], gastric [11], intraocular [12], breast [13], lung [14], and skin cancer [15].

Despite its beneficial effects, curcumin’s clinical applications are restricted due to its poor bioavailability, very low aqueous solubility, and chemical instability at physiological pH [16]. To resolve this issue, several formulation strategies have been investigated to enhance curcumin bioavailability, including lipid additions, the adsorption and dispersion of curcumin on a wide range of matrices, and particle size reduction-based techniques [17]. Furthermore, several investigations were reported to enhance curcumin aqueous solubility, expand its stability, and improve its bioavailability, such as liposomal formulations and biopolymeric nano-complexation [18,19,20,21,22]. In addition, the complexation of curcumin with β-cyclodextrin is considered a practical approach to improve water solubility, counteract curcumin degradation, and sustain curcumin release [23].

Cyclodextrins are not toxic when taken orally as they are not absorbed in the upper GI tract and are digested by colonic bacteria. In addition, they have been classified as GRAS (Generally Recognized as Safe) substances [24]. β-Cyclodextrin is a macrocyclic oligosaccharide that consists of seven glucopyranose units connected by α-(1,4) glucosidic linkage, resulting from glucopyranose units’ chair conformation of cyclodextrin shaped like a cone with dual open ends, with an inner diameter of 7.8 Å and outer diameter of 15.3 Å [25,26]. β-Cyclodextrin is sparingly soluble in water [27]; hydrogen bonding is considered the key role of cyclodextrin solubility in aqueous media [25]. β-Cyclodextrin has a molar mass of 1135 Dalton with a calculated Log P of −14.82 and a polar surface area value of 554.05 [25]. 

In the literature, several studies show that curcumin–cyclodextrin inclusion complexes can be used for pharmaceutical applications. The formation of cyclodextrin-based inclusion complexes can enhance and sustain drugs’ stability [28] and improve their aqueous solubility [29,30,31]. The formation of cyclodextrin-based inclusion complexes was recently proven to enhance the solubilities and thermal stabilities of difenoconazole, thiophanate, and thiram and improve the physical characteristics of environmentally safe cyclodextrin-based nanofiber pesticidal formulations [32,33,34,35]. A recently published study reported a 206-fold improvement in curcumin solubility and proved prolonged curcumin release from the resulting inclusion complexes [23]. Sharma et al. established the prospect of the investigation of the curcumin-β-cyclodextrin complex to be electro sprayed on biomedical outsides of implantation and devices to increase effectiveness and further improve curcumin stability [36].

Cyclodextrins can be cross-linked using various multifunctional chemical reagents, including carbonyl compounds, dianhydrides, carboxylic acids, diisocyanates, and epoxides, to produce a nanosponge formulation [37]. The method of nanosponge synthesis was comprehensively reviewed for pharmaceutical applications [38,39,40]. 

Nanosponge is a nanoporous carrier with a sponge-like structure formed through the hyper cross-linking of cyclodextrin polymers to form three-dimensional covalent systems with nanochannels. Nanosponge is an attractive drug carrier to be considered for pharmaceutical applications [41,42], due to its stability, insolubility, biocompatibility, and ability to encapsulate drugs through the formation of inclusion and non-inclusion complexes. In addition, nanosponge has been reported to enhance the solubility of different drugs [43,44,45], enhance the permeation and retention of dermal formulations [43,46], control the rate of drug release [47,48], increase drug stability and lessen drug degradation [47,49], and help mask the drug’s unpleasant taste [50]. 

The inclusion complex of curcumin with cyclodextrin polymer [23,51,52,53,54] and with cyclodextrin-based nanosponges [55,56,57,58,59,60] was studied thoroughly in previously published research and was reported to have an effect on the enhancement of curcumin solubility, release rate, stability, and cytotoxicity [55,56,57,58,59,60,61]. However, there was no comparison between nanosponges and direct β-cyclodextrin complexations for curcumin. Therefore, this study explores the complexation efficiency of the curcumin complex with β-cyclodextrin compared to β-cyclodextrin-based nanosponge. 

**Figure 1:**
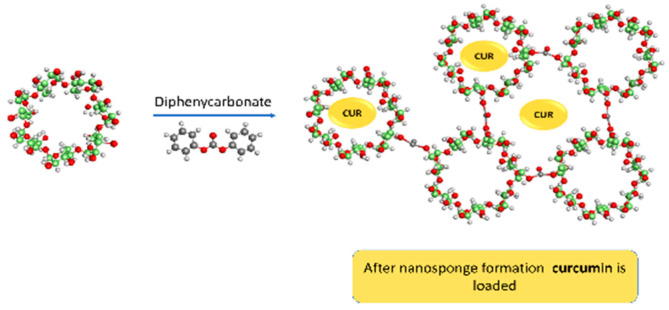
Schematic representation of the curcumin-loaded DPC cross-linked nanosponges.

Figure 1 shows the schematic representation of curcumin-loaded cyclodextrin-based nanosponges.

## 2. Materials and Methods

### 2.1. Materials

Curcumin and β-cyclodextrin polymer were purchased from Sigma Aldrich chemical (Louis, MO, USA) and HPLC grade water and absolute ethanol were purchased from (Honeywell, France). Diphenycarbonate (DPC) was provided by TCI Chemicals (Tokyo Chemical Industry Co., Ltd., Tokyo, Japan), and acetonitrile HPLC grade was purchased from Scharlau (Barcelona, Spain), Acetone HPLC grade Lab Chem (Zelienople, PA, USA) Potassium dihydrogen phosphate was supplied by AZ Chem for chemicals (Thunder Bay, ON, Canada). Potassium phosphate dibasic was provided by Xilong Chemical industry (Shantou, Guangdong, China). β-Cyclodextrin polymer was dried in a vacuum oven at 40 °C until a constant weight was obtained. All other materials were used as supplied without any further modifications. 

### 2.2. Methods

#### 2.2.1. Preparation of Plain Cyclodextrin DPC Cross-Linked Nanosponge

Cyclodextrin nanosponge was synthesized by utilizing a solvent-free melting method [62]. Briefly, accurately weighed amounts of β-cyclodextrin and DPC cross-linker were melted inside a round-bottom flask heated inside a paraffin oil bath on a hot plate at 90 °C, with continuous magnetic stirring for five hours. The resulting mass was crushed and repeatedly washed with ultrapure water, then extracted with absolute acetone in triplicate to get rid of the phenol byproduct, and then allowed to dry at room temperature. Finally, 1 mM of the β-cyclodextrin polymer was mixed with 4, 6, or 8 mM of DPC cross-linker to prepare NS4, NS6, and NS8, respectively.

#### 2.2.2. Preparation of the Drug-Loaded Nanosponges

Curcumin was loaded in the prepared plain nanosponges. In brief, a different weight ratio of the plain nanosponge and the drug was weighed and dissolved in 20% ethanol in ultrapure water; the mixture was sonicated for 10 min, then stirred for 24 h in the dark at a constant room temperature; the resulting dispersions were centrifuged at 2000 rpm for 15 min and then the supernatants were freeze-dried for 72 h.

#### 2.2.3. Curcumin-β-Cyclodextrin Complex

Excess curcumin was added to an aqueous solution of 8 mM β-cyclodextrin raw polymer dispersion sonicated for 15 min; the mixture was then shaken at 100 rpm for 24 h at 25 °C in a thermostatic water bath. Later, the resulting dispersions were centrifugated at 3500 rpm for 10 min. Finally, the supernatant was dried using a freeze drier.

#### 2.2.4. Corresponding Physical Mixtures

Curcumin and β-cyclodextrin physical blend was made by mixing raw curcumin and raw β-cyclodextrin polymer. Meanwhile, nanosponge corresponding physical mixtures were prepared by mixing raw curcumin and plain nanosponges.

#### 2.2.5. HPLC Analysis of Curcumin Concentration

The high-performance liquid chromatography RP-HPLC method was used for curcumin concentration determination [63]. LC-2030 HPLC system (Shimadzu Corporation, Kyoto, Japan) was used. Curcumin was assayed using a UV LC-2030/2040 PDA Detector set at a wavelength of 426 nm. Low-pressure gradient LC-2030 Pumps were used for mobile phase elution where the mobile phase ingredients were composed of a buffer of pH 5: acetonitrile in a ratio of (45:55 *v*/*v*) on a rate of 1.3 mL/minute, samples was directly injected into the HPLC system, and samples were separated using Thermo Scientific Hypersil ODS C18 column (250 mm × 4.6 mm, and 5 μm particle size); with an oven temperature of 33 °C, linearity was detected in the range of 1.5–100 μg/mL and was obtained with R = 0.99996. Data analysis was performed utilizing Shimadzu LabSolutions software (Shimadzu Corporation, Kyoto, Japan).

#### 2.2.6. Phase Solubility Studies

Phase solubility experiments are commonly used to assess the complexation potential to increase substrate aqueous solubility. It allows a better understanding of the formed complex by calculating several important parameters, including the stability constant and complexation efficiency. In addition, it allows fast estimation of cyclodextrin solubilizing efficacy and the optimum molar ratio between cyclodextrin polymer and the studied substrate. Thus, it plays a vital role in the evaluation of cyclodextrin use in pharmaceutical formulations [64]. 

Curcumin is unstable in aqueous media; it hydrolytically degrades to Trans-6-(4′-hydroxy-3′-methoxyphenyl) with time 2,4-dioxo-5-hexenal, vanillin, ferulic acid, and feruloyl methane [65]. Therefore, Mondal et al. studied curcumin degradation rates in various diluents and solutions in aqueous media, utilizing UV visible studies and steady-state fluorescence spectral experiments. The study results indicated that the presence of organic solvents enhances the stability of curcumin. Among the studied solvents, ethanol offered the best stability of curcumin, as the reported degradation rate constants of curcumin at 30 °C were 15.7 and 1.17 decay/day in water and 20% ethanol, respectively [66]. For this reason, a 20% (*v*/*v*) ethanolic solution was used in this work for phase solubility studies. 

A phase solubility study for curcumin complexation with cyclodextrin polymers was conducted previously in the presence and absence of 10% (*v*/*v*) ethanol. The study results suggested that the addition of ethanol had no significant alteration to the phase solubility profile shape. Even though certain variations in solubility were detected, the addition of ethanol allowed the detection of the intrinsic solubility of curcumin [67].

Phase solubility studies were carried out inconsistent with the method of Higuchi–Connors [68]. In brief, an excess amount of curcumin was added to a series of aqueous solutions containing an increased level of β-cyclodextrin (0 to 100 mM) and β-cyclodextrin nanosponges (0–2%). Mixtures were continuously shaken at 100 rpm in the dark at a constant 25 °C temperature inside the thermostatic water bath shaker, GFL, Germany, for 24 h, then centrifuged. The supernatant’s curcumin concentrations were determined using the HPLC method. All experiments were repeated in triplicate.

(1) $$\mathrm{Ks}=\frac{\mathrm{slope}}{S{}^{\circ}\;\left(1-\mathrm{slope}\right)}$$

The slope obtained from the linear part of the phase solubility diagram, S°, is curcumin’s intrinsic solubility in the absence of cyclodextrin polymer or nanosponge.

Complexation efficiency, which is equivalent to the formed complex: free β-CD ratio, is calculated using the following equation:

(2) $$\mathrm{CE}=\frac{\left[\beta \mathrm{CD}/\mathrm{Guest}\right]\;}{\left[\beta -\mathrm{CD}\right]\;}=\frac{\mathrm{slope}}{1-\mathrm{slope}}$$

(β-CD/guest) is the level of the dissolved complex, and (β-CD) is the level of free β-CD. Accordingly, the complexation efficiency value allowed the estimation of the curcumin: β-CD optimum ratio using the following equation [69]:

(3) $$\mathrm{Guest}:\;\beta \mathrm{CD}=1:\left(1+\frac{\mathrm{slope}}{1-\mathrm{slope}}\right)$$

#### 2.2.7. Molecular Modeling Studies

Molecular modeling was utilized in this study to investigate the probable complexation mechanism of curcumin-β-cyclodextrin. Several schemes for curcumin–cyclodextrin complexation have been proposed in the literature. The first scheme proposed that the mechanism of curcumin-β-cyclodextrin host–drug inclusion complexation proceeds with a 2:1 stoichiometric ratio, in which each of the two aromatic rings of curcumin is interacting with and is included within the wide rim of one molecule of β-cyclodextrin via hydrogen bonding [70,71], while the other proposes that the complexation scheme proceeds with a 1:1 ratio [67,71,72,73,74]. 

The two possible schemes were investigated using molecular docking methods. Hence, the 3D structure of β-cyclodextrin was obtained from the Protein Data Bank (https://www.rcsb.org/, (accessed on 9 November 2021).) (PDB ID 3M4E), and that of curcumin was downloaded from the PubChem database (https://pubchem.ncbi.nlm.nih.gov/, (accessed on 9 November 2021)). All modeling steps were performed using Biovia Discovery Studio (DS) 2020 (BIOVIA Discovery Studio-BIOVIA-Dassault Systèmes^®^ (3ds.com, (accessed on 9 November 2021))). First, two molecules of β-cyclodextrin were manually aligned face-to-face (wide rims of the two β-cyclodextrin molecules were pointing inward) and were defined as a receptor with a sphere of 14.8 Å radius using the Define and Edit Binding Site in DS. Then, curcumin was docked into the defined receptor using the CDOCKER docking protocol in DS. For the other scheme, one β-cyclodextrin molecule was defined as a receptor with a sphere of 11 Å radius, and curcumin was docked into it. Default parameters for CDOCKER were used, where 10 random starting conformations were generated from equilibration and minimization of the starting curcumin structure; 10 diverse top docked poses were saved after being refined using simulated annealing. 

#### 2.2.8. Physicochemical Evaluation of Curcumin-β-Cyclodextrin Complex and Curcumin-Loaded Nanosponge

The formulated complex and nanosponges were characterized to ensure the formation of an inclusion complex of curcumin with cyclodextrin polymer and in cyclodextrin-based nanosponge.

##### Particle Size Distribution and Polydispersity Index

Freshly prepared formulations were diluted with deionized water, and the particle size and size distribution were measured using a Malvern zeta sizer, Malvern Panalytical Ltd., Malvern, UK, at 25 °C. 

##### Zeta Potential

The sample’s zeta potentials in aqueous suspensions of a concentration were analyzed at 25 °C using a Malvern zeta sizer, Malvern Panalytical Ltd., Malvern, UK, with a 173° detection angle to increase the sensitivity of dynamic light scattering [75]. 

##### The Powder Diffraction Pattern (PXRD)

The X-ray powder diffractometer, Ultima IV diffractometer, Rigaku, Japan, was used to record the X-ray diffraction patterns for curcumin, curcumin-β-cyclodextrin complex, raw β-cyclodextrin polymer, curcumin-loaded nanosponge, and plain nanosponge. 

##### Differential Scanning Calorimetry (DSC)

DSC thermograms of curcumin, curcumin-β-cyclodextrin complex, raw β-cyclodextrin polymer, curcumin-loaded nanosponge, and plain nanosponge were conducted using Differential Scanning Calorimeter DSC 204 F1 Phonex, Netzch, Germany. Samples were placed into sealed aluminum pans. A temperature range program was performed between 30 and 400 °C under a continuous nitrogen flow rate of 10 °C/min. 

##### Fourier-Transform Infrared (FTIR) Spectroscopy 

FTIR spectra were conducted using the Fourier transform infrared spectroscopy model, IRAffinity-1, Shimadzu, Japan, with a resolution value of 4 cm^−1^, in the range of 400–4000 cm^−1^, for curcumin, curcumin-β-cyclodextrin complex, raw β-cyclodextrin polymer, curcumin-loaded nanosponge, plain nanosponge, and corresponding physical mixtures. Before measurement, the powder was mixed with KBr and finely ground using a mortar and pestle.

##### In Vitro Release Studies

Accurately weighed samples containing an equivalent amount of 1 mg curcumin were suspended in 3 mL of release medium and placed in 7 cm long dialysis membrane tubes (Visking, molecular weight cutoff 12–14,000 Daltons, Medicell Membrane Ltd., London, UK), closed from both sides, inserted in a preheated 100 mL of the ethanol: phosphate buffer of pH 7.4 (1:1) inside a well-closed glass bottle, in a water bath shaker at a constant temperature of 37 °C, and rotated at 100 rpm. The dialysis membrane diffusion method was used as previously described [76]. At determined time intervals, 1 mL aliquots were withdrawn and replaced with an equivalent volume of release medium, and the whole release medium was changed with fresh medium at a predetermined time to obtain sink condition. Curcumin concentration was determined using the HPLC method described in the phase solubility study. In vitro release study analyzed raw curcumin, the formed curcumin complexes, and the corresponding physical mixtures. All experiments were repeated in triplicate.

##### Scanning Electron Microscopy (SEM)

The morphology of the prepared nanosponge was observed using a scanning electron microscope, Jeol-JSM-5300 scanning electron microscope, Tokyo, Japan.

#### 2.2.9. Statistical Analysis

To assess whether the differences between raw curcumin complex and nanosponge are more than expected by chance, repeated measures two-way ANOVA statistical analysis was conducted using GraphPad Prism 9.0.0, with an alpha level of 0.05.

## 3. Results and Discussion

### 3.1. Phase Solubility Studies

**Figure 2:**
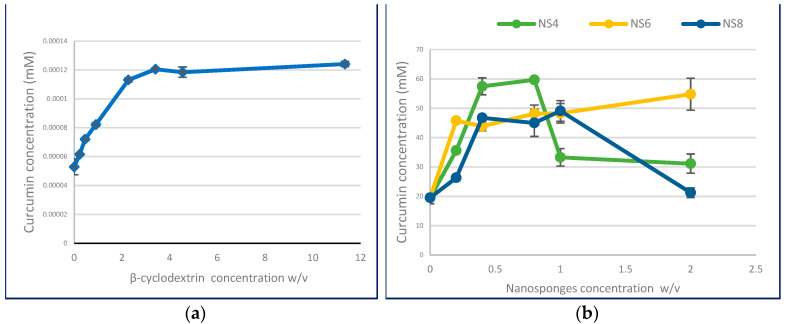
Phase solubility diagrams of: (**a**) curcumin-β-cyclodextrin complex; (**b**) curcumin-loaded nanosponges.

The solubility of curcumin increases as a feature of β-cyclodextrin level increases, displaying the AN type of solubility phase profile (Figure 2a). Meanwhile, the curcumin complex with nanosponge exhibits the Bs type of solubility phase diagram, as shown in Figure 2b. It comprises three regions: in the first part, curcumin solubility increases with the increase in nanosponge concentration resulting from the complex formation. Then, further increments in nanosponge concentration lead to complex precipitation, as shown in the solubility phase diagram results.

Table 1 represents the determined parameters of the complex formation of curcumin with β-cyclodextrin and β-cyclodextrin-based nanosponges. These parameters are calculated according to the slope of the resultant initial linear part of the solubility phase diagram; all regression lines show a linear correlation (R^2^ ≥ 0.994).

Total curcumin solubility was enhanced by the formation of β-cyclodextrin inclusion complexes up to 2.34-fold compared to the inherent solubility and increased for curcumin nanosponges up to 2.95, 2.55, and 2.51-fold for nanosponge NS4, NS6, and NS8, respectively. At the same time, the determined Kc of the curcumin complex with β-cyclodextrin was 487.34 M^−1^. Meanwhile, Kc values increased remarkably for curcumin nanosponges compared to β-cyclodextrin, where the calculated kc values were 4972.90, 4164.50, and 3567.87 M^−1^ for nanosponge NS4, NS6, and NS8, indicating a decrease in the stability constant with the increase in cross-linking density. In addition, results indicate that complexation efficiency also increased for nanosponge formulation and decreased with increasing cross-linking density.

The mean value of the stability constant of the β-cyclodextrin (1:1) complex with different substrates was reported in the literature to be around 490 M^−1^ [77]. While previous results for curcumin complexation showed variability in different study conditions, the stability constant value was reported as 198 M^−1^ in deionized water at room temperature [78]. An A_L_ type phase solubility profile was reported with a stability constant value of 167 M^−1^ at 30 °C in sterile water with a stoichiometric ratio of (1:1) [71]. Another study reported a Bs type solubility phase diagram with a stoichiometric ratio of (2:1) and a stability constant value of 1457 M^−1^; the study was conducted in distilled water and equilibrium solubility was achieved after 5 days of the experiment, and the effect of curcumin hydrolytic degradation of curcumin during the solubility experiment was not specified in the study. However, the study reported that the formation of the inclusion complex reduced curcumin degradation in aqueous media for 8 h to percentage degradation values ranging between 20 and 40% compared to more than 70% degradation of pure curcumin [79].

In contrast to our findings, a recent study reported an increase in the complexation stability constant and solubility enhancement for sulfamethoxazole interaction with cyclodextrin-based nanosponges with an increase in the cross-linker molar ratio at the studied range of cyclodextrin: cross-linker molar ratio of (1:2–1:4) [80].

Castiglione et al.’s study proved that the density of cyclodextrin polymer cross-linking increased with the increment of the molar ratio of the carbonate cross-linker at the studied range of molar proportions of β-cyclodextrin to cross-linker from (1:2–1:8) [81]. Another study by Rossi et al. discussed the networking properties of cyclodextrin nanosponge using Raman and Brillouin scattering experiments. The study investigated the effect of the cross-linking ratio on the resulting nanosponge’s mechanical properties, which can reflect their swelling and entrapment ability. The results proved that the increase in the cross-linker ratio would increase the cross-linking of the cyclodextrin polymer to a specific limit, after which saturation is achieved due to steric effect. Any further increase in the cross-linker amount could not increase polymer cross-linking further; instead, it would allow cyclodextrin unit branching. This effect can vary depending on the type of cross-linker, as saturation is achieved at a molar ratio of (1:2) when β-cyclodextrin is cross-linked by carbonyldiimidazole.

Meanwhile, saturation reached a molar ratio of (1:6) when using the cross-linker pyromellitic dianhydride [82,83], and EDTA dianhydride [84]. In addition, the higher ratio of cross-linking agents may be attributed to the excess amount of cross-linkers in the prepared nanosponge [85], limiting the access of curcumin to nanosponge channels, which restricts the ability of curcumin to enter the binding sites in nanosponge. This explains the decreased complexation with increasing cross-linking density. Further studies such as NMR analysis of the complexations are required to further comprehend the complexation of curcumin with cyclodextrin and cyclodextrin-based nanosponge. 

The nanosponges were prepared by mixing the cyclodextrin powder with the already molten DPC; preliminary trials were performed for the preparation of cyclodextrin-based nanosponge with a lower cross-linking density. By visual observation, the use of lower ratios of DPC than 1:4 means the quantity of molten DPC cannot wet the added cyclodextrin powder, a considerable amount of cyclodextrin powder remains in solid separated status, and that excess powder is not mixed with DPC nor involved in the reaction.

### 3.2. Molecular Modeling

Molecular simulations of the cyclodextrin complex can represent the formed complex at the atomic level [86]. Therefore, the two proposed mechanisms of curcumin-β-cyclodextrin host–drug inclusion complexation were investigated using molecular docking studies. The docking results showed that both complexation schemes have comparable binding affinities. For the first proposed scheme, a 1:2 stoichiometric ratio of curcumin-β-cyclodextrin, the docking scores revealed the binding energy of curcumin ranging from −19.5 to −16.98 kcal/mol, and for the second scheme, a 1:1 ratio, the curcumin binding energy ranged from −23.5 to −20.2 kcal/mol. Given the relative binding energy values of the two complexation schemes, it seems that both complexation possibilities are likely. However, the binding interactions in the 1:1 complex seem to be more favorable. 

**Figure 3:**
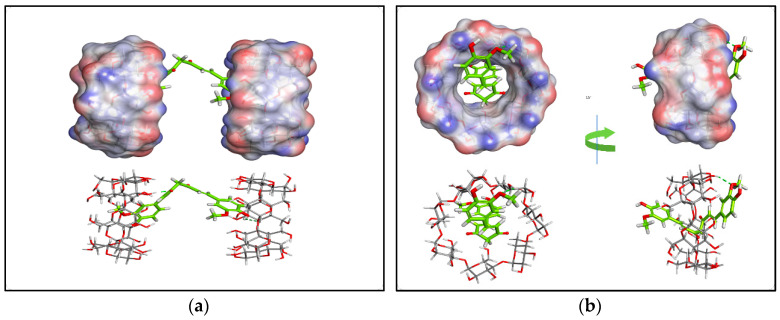
The binding orientation of curcumin in both complexation schemes with β-cyclodextrin. (**a**) The complexation scheme involves a 1:2 ratio of curcumin: β-cyclodextrin; (**b**) The second possible complexation scheme involves a 1:1 ratio. The upper panels show the surface representation of β-cyclodextrin, while curcumin is shown as sticks. In the lower panels, β-cyclodextrin is shown as sticks to enable better visualization of intermolecular hydrogen bonds (dashed green lines).

Figure 3 shows the binding orientation of curcumin in both complexation schemes. Again, the interaction seems to be mainly related to hydrogen bonding.

### 3.3. Zeta Potentials and Particle Size Distribution

Particle size examination and zeta potentials are illustrated in Table 2. The results show that the curcumin-β-cyclodextrin complex has an average particle size of 6.76 ± 1.76 µm with a polydispersibility index (PDI) of 0.20 ± 0.02. The large particle size of β-cyclodextrin-curcumin complexes can be attributed to the high tendency for aggregate formation that is not easily redispersed upon contact with water. On the other hand, curcumin-loaded nanosponge NS4 has an average particle size of 266.60 ± 15.84 nm with a polydispersibility index (PDI) of 0.27 ± 0.02. Furthermore, the zeta potential of the curcumin-β-cyclodextrin complex exhibited two sharp peaks (Figure S2) at 14.60 ± 2.33 (64.8%) and −25.90 ± 3.53 (35.3%); meanwhile, curcumin-loaded nanosponge NS4 was found to have a negative zeta potential of −21.57 mV.

The negative surface of cyclodextrin signifies the molecular positioning of cyclodextrin in a way that the hydroxyl groups are mainly directed towards the surrounding aqueous media, and the complexation of curcumin with cyclodextrin polymer could block these hydroxyl groups, and this expected decrease in zeta potential was experimentally established and reported by Rachmawati et al. as the increase in the curcumin amount decreases the negative surface charge from −39.6 to −17.3 by increasing the curcumin amount from 5% to 20% [87]. 

A curcumin-loaded nanosponge particle size was reported previously for dimethyl carbonate cross-linked cyclodextrin with an average of 487.3 nm (PDI = 0.476), with a zeta potential value of −27 mV [45]. 

### 3.4. The Powder Diffraction Pattern (PXRD)

**Figure 4:**
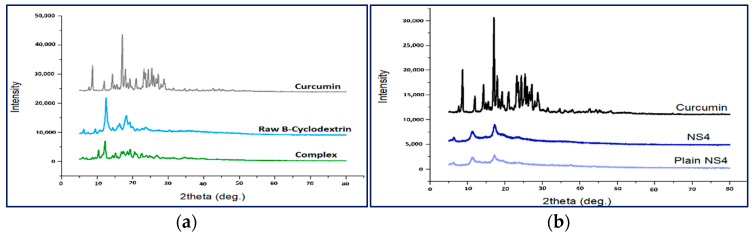
PXRD patterns of (**a**) curcumin-β-cyclodextrin complex; (**b**) curcumin-loaded nanosponges compared to the raw materials.

The characteristic peaks of curcumin (Figure 4) appear at 8.62°, 11.9° and 14.2°, 17.02° (2θ). Similar peaks were previously reported in the literature [88]. Furthermore, according to previous research, these peaks can be related to crystalline curcumin polymorph Form I [89].

The PXRD pattern of the curcumin complex with β-cyclodextrin showed the disappearance of curcumin characteristic peaks (Figure 4a), and the appearance of new weak peaks in the diffractogram of the complex indicates the formation of an inclusion complex existing as a new crystalline phase. Similar findings were previously reported for the complexation of curcumin with β-cyclodextrin polymer using the co-precipitation method [90]. 

The PXRD pattern of the curcumin-loaded nanosponge also displayed the disappearance of curcumin characteristic peaks (Figure 4b), confirming the encapsulation of curcumin in the nanosponge.

### 3.5. Differential Scanning Calorimetry (DSC)

**Figure 5:**
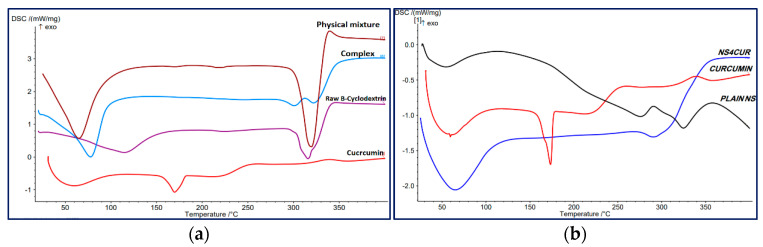
DSC thermograms of (**a**) curcumin-β-cyclodextrin complex; (**b**) curcumin-loaded nanosponges compared to the raw materials.

The DSC thermogram of curcumin (Figure 5) showed an endothermic peak at 173.3 °C, representing the melting point of curcumin. The melting point of curcumin in the literature was reported in the range of (172.85–187) °C [78,89,91,92]. 

The thermogram of β-cyclodextrin (Figure 5a) showed two endothermic peaks: the broad peak at 115.3 °C represents water evaporation, and the peak at 312.7 °C represents the melting point of the β-cyclodextrin polymer [92].

The DSC thermogram of the curcumin complex with cyclodextrin displayed the disappearance of the curcumin endothermic peak. This indicates the encapsulation of curcumin in the cavity of β-cyclodextrin polymer substituting water molecules and confirms the inclusion complex formation; similar findings were reported in the literature [78,91,92].

The DSC thermogram of curcumin-loaded nanosponge compared to the plain nanosponge and curcumin is represented in Figure 5b, and the disappearance of the curcumin endothermic peak indicates the formation of the inclusion complex and the encapsulation of curcumin inside the prepared nanosponges. Similar results were reported in the literature [56,60].

### 3.6. Fourier-Transform Infrared Spectroscopy

**Figure 6:**
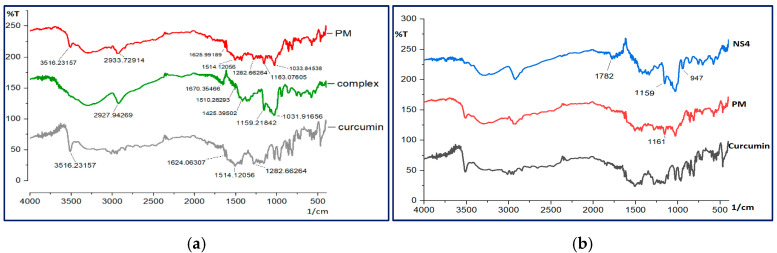
FTIR spectra of (**a**) curcumin-β-cyclodextrin complex; (**b**) curcumin-loaded nanosponge compared to the raw curcumin and the corresponding physical mixtures.

In Figure 6a, the representative peaks of the curcumin complex with β-cyclodextrin, raw curcumin, and the corresponding physical mixture are displayed. This demonstrated the representative peaks of curcumin at 3516 cm^−1^ attributed to the O–H stretching vibration, 1624 cm^−1^ indicative of benzene ring stretching vibration, 1514 cm^−1^ indicative of the presence of mixed (C–C) and (C–O) vibrations and (C=O) carbonyl bond vibrations and in-plane bending vibrations around aromatic (CC–H) of keto and enol configuration forms of curcumin, 1427 cm^−1^ representing the aromatic (C=C) stretching vibration, and 1262 cm^−1^ representing the (C–O) bending vibrations of the phenolic band. Similar findings were reported in the literature [78,93,94].

The FTIR spectrum of the physical mixture displayed the characteristic bands of curcumin beside the characteristic peaks of the cyclodextrin polymer at 2933 cm^−1^ corresponding to the (CH2) group stretching vibration, and at 1033 cm^−1^ representing the (C–O–C) stretching vibration, in agreement with the literature [78]. 

The FTIR spectrum of the curcumin complex with β-cyclodextrin proved the disappearance of curcumin’s characteristic peaks. In contrast, β-cyclodextrin characteristic peaks can be found, which offers a considerable indication of inclusion complex formation; similar findings were reported previously [78]. Furthermore, FTIR analyses were carried out for curcumin-loaded nanosponge (Figure 6b) compared to curcumin and the corresponding physical mixture, which displayed the absence of the curcumin characteristic peak, which suggests the encapsulation of curcumin inside the prepared nanosponge. A similar finding was reported previously [85]. The absence of curcumin characteristic peaks in both the curcumin complex with β-cyclodextrin and curcumin-loaded nanosponge is attributed to the successful formation of the curcumin inclusion complex with cyclodextrin polymer.

### 3.7. In Vitro Release Experiments

**Figure 7:**
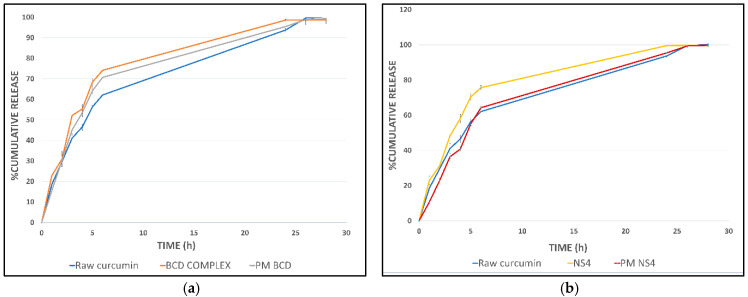
In vitro release profiles of (**a**) curcumin-β-cyclodextrin complex (BCD complex); (**b**) curcumin-loaded nanosponges (NS4) compared to the raw curcumin and the corresponding physical mixtures (PM BCD and PM NS4).

The in vitro release profile of the curcumin complex with β-cyclodextrin is presented in Figure 7a; curcumin’s complexation enhances curcumin release. The difference in release rate was less than anticipated from the solubility enhancement. This can be related to adding ethanol to the dissolution media to stabilize curcumin in the aqueous media. The release profile of the corresponding physical mixture was comparable to the complex. This can be related to the rapid formation of the complex upon exposure to the dissolution media. An enhancement in the curcumin release rate from the cyclodextrin complex was stated previously [23,95,96,97].

The in vitro release profile of curcumin-loaded nanosponge showed an enhancement in curcumin release, as displayed in Figure 7b, and curcumin was released from the NS4 sample faster than from the physical mixture and the raw curcumin. In addition, an enhancement in the release rate of curcumin from cyclodextrin-based nanosponge was reported by Pushpalatha et al. [56]. However, the statistical analysis results disapprove of significant differences between the evaluated release profiles, with P values greater than 0.05. On the other hand, the in vitro release of curcumin from the NS4 sample did not show any significant difference from the cyclodextrin complex as shown in (Figure S1).

Drug release kinetics studied by fitting the release profile for the first 6 h on the empirical release models, which represent the release of more than 70% of the drug for both the cyclodextrin complex and prepared nanosponge, showed that curcumin-loaded nanosponge (NS4) best fit to the zero-order model with an R value of 0.991; meanwhile, the cyclodextrin complex best fit with the first-order release kinetic with R = 0.998.

The determined amount of curcumin in the characterized dried β-cyclodextrin complex was 1.2 ± 0.14% *w*/*w*; meanwhile, the contents of curcumin in the loaded nanosponge were 1.52 ± 0.25, 1.52 ± 0.26, and 2.62 ± 0.12% *w*/*w* for NS4, NS6, and NS8, respectively.

Further studies are required to demonstrate that curcumin was encapsulated and then released from the prepared formulations and retained antioxidant activity.

### 3.8. Scanning Electron Microscopy (SEM)

**Figure 8:**
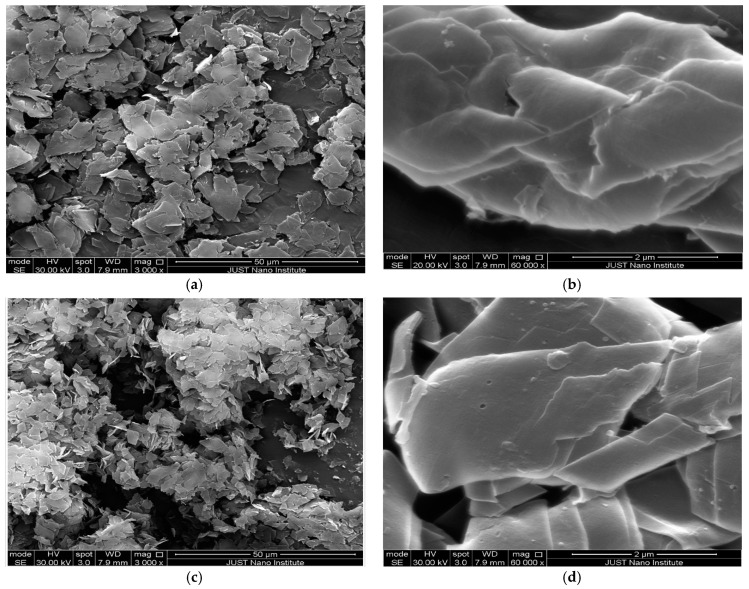
SEM images of: (**a**) curcumin-loaded DPC cross-linked nanosponge (3000×); (**b**) curcumin-loaded DPC cross-linked nanosponge (60,000×); (**c**) curcumin-β-cyclodextrin complex (3000×); (**d**) curcumin-β-cyclodextrin complex (60,000×).

Figure 8a,b show the SEM images of curcumin-loaded DPC cross-linked nanosponges in a molar ratio of 1:4 (sample NS4); as seen from the images, the flake-like crystalline nature of the prepared nanosponge was observed. The results are in agreement with the previously published study [60]. Figure 8c,d display the SEM images of the curcumin-β-cyclodextrin complex; the images demonstrate the crystalline rhomboid-shaped morphology without any visible cracks or fractures, suggesting the formation of the inclusion complex of drug with cyclodextrin [98]. SEM images were conducted for the dried samples. Meanwhile, particle size measurements were conducted in aqueous dispersions. This explains the difference between the SEM images and the resulting particle size distribution, as the particles are most probably aggregated together in the solid state and redispersed in contact with water.

## 4. Conclusions

β-Cyclodextrin cross-linking enhanced the complexation efficiency and solubilization effect of β-cyclodextrin polymer, indicating the potential of utilizing nanosponge formulation for pharmaceutical application for poorly soluble active ingredients. This study proved the successful formation of curcumin in cyclodextrin complexes when equilibrated in a 20% ethanolic solution and compared the complexation stabilities of curcumin inclusion complexes with β-cyclodextrin related to the cross-linked cyclodextrin nanosponges. Compared to the curcumin-β-cyclodextrin complex, curcumin in cross-linked β-cyclodextrin nanosponges resulted in a more significant enhancement in drug solubility and increased the complexation stability. The study results also indicated a decrease in the complexation efficiency and solubilization effect with the increase in the cross-linker amount. They indicated the negative effect of further increasing the molar ratio of diphenyl carbonate by more than 1:4 of β-cyclodextrin: cross-linker. 

## Figures and Tables

**Table 1:** Regression line equation and coefficient, complexation constant (Kc), and complexation efficiency (CE) as obtained from the linear part of the phase solubility diagram.

	Equation	R^2^	Kc (M^−1^)	Complexation Efficiency	Ratio ^1^	Type of Curve
β-Cyclodextrin	Y = 0.0258X + 0.0561	0.994	487.34	0.03	1:1	AN
NS4	Y = 0.2634X + 0.0477	0.994	4972.90	0.26	1:1	BS
NS6	Y = 0.2206X + 0.0522	0.998	4164.50	0.22	1:1	BS
NS8	Y = 0.1891X + 0.0512	0.997	3567.87	0.19	1:1	BS

^1^ Calculated depending on the linear line of the solubility phase diagram.

**Table 2:** Zeta potentials, particle size, and size distribution of the prepared complexes.

	Zeta Potential	SD	Size (nm)	SD	PDI	SD
**β-Cyclodextrin Complex ^1^**	14.6 ± 2.33 (64.8%) −25.9 ± 3.53 (35.3%)	2.25	6759	1762.11	0.201	0.015
**NS4**	−21.57	4.85	266.6	15.84	0.321	0.044
**Raw β-Cyclodextrin**	−29.10	5.37	3514	2554.07	0.533	0.455
**Plain NS4**	−18.30	4.67	85.74	1.99	0.265	0.021
**Raw Curcumin**	−24.2	2.25	5956.5	968.03	0.493	0.169

^1^ Two peaks appeared for the Zeta potential of the β-cyclodextrin complex.

## Data Availability

The data presented in this study are available on request from the corresponding author.

## Supplementary Materials

The following are available online at https://www.mdpi.com/article/10.3390/polym13234073/s1, Figure S1: In vitro release profile of curcumin-β-cyclodextrin complex compared to curcumin-loaded nanosponge. Figure S2: Zeta potentials of (**A**) raw β-cyclodextrin, (**B**) curcumin-β-cyclodextrin complex, (**C**) plain NS4, (**D**) curcumin-loaded NS4, and (**E**) raw curcumin.
