# Supplementary material for: Evaluation and Characterization of Curcumin-β-Cyclodextrin and Cyclodextrin-Based Nanosponge Inclusion Complexation
Source: Polymers (Basel). 2021 Nov 24;13(23):4073. doi: 10.3390/polym13234073 (PMC8658939; doi:10.3390/polym13234073)
Supplement: Supplementary file 1 [file polymers-13-04073-s001.zip › polymers-1415666-supplementary.pdf]

**Supplementary Materials:**

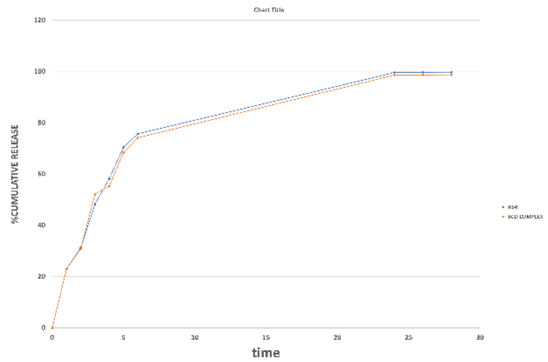


**Figure S1.** In vitro release profile of curcumin–β-cyclodextrin complex compared to curcumin-loaded nanosponge.

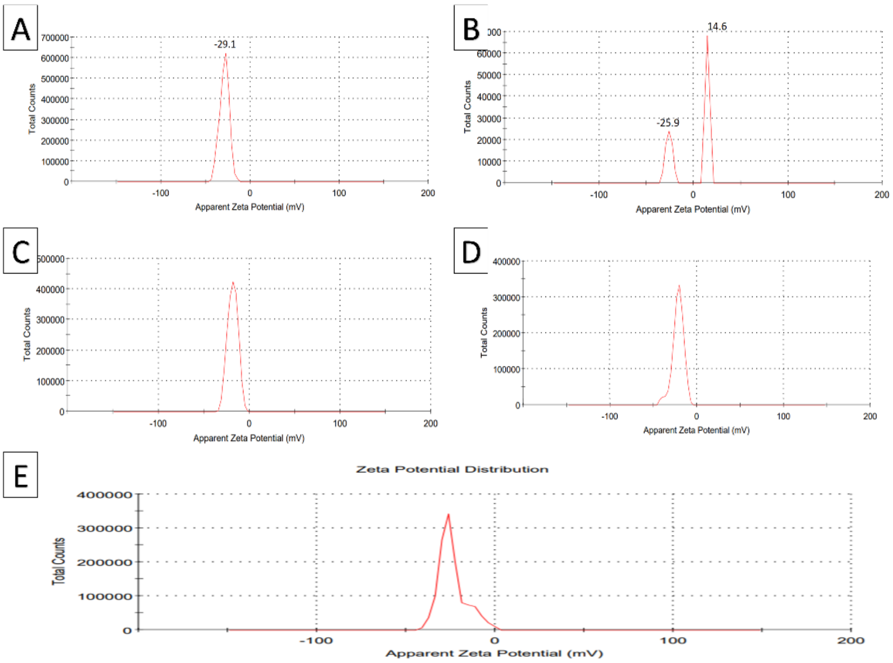


**Figure S2.** Zeta potentials of (**A**) raw β-cyclodextrin, (**B**) curcumin–β-cyclodextrin complex, (**C**) plain NS4, (**D**) curcumin-loaded NS4, and (**E**) raw curcumin.
